# Effect of assistive devices on the precision of digital impressions for implants placed in edentulous maxilla: an in vitro study

**DOI:** 10.1186/s40729-021-00397-w

**Published:** 2021-12-13

**Authors:** Rena Masu, Shinpei Tanaka, Minoru Sanda, Keita Miyoshi, Kazuyoshi Baba

**Affiliations:** Department of Prosthodontics, Showa University School of Dentistry, 2-1-1 Kitasenzoku, Ota-ku, Tokyo, 145-8515 Japan

**Keywords:** Digital dentistry, Digital impression, Intraoral scanner, Dental implants, Precision

## Abstract

**Purpose:**

To examine the effect of assistive devices on the precision of digital impression for multiple implants placed in the edentulous maxilla.

**Methods:**

A reference model representing an edentulous maxilla with four implants was developed. The digital impression group included three settings: Type 0, without an assistive device; Type 1, with an assistive device connecting only neighboring implants; and Type 2, with an assistive device connecting not only neighboring implants but also the two posterior implants, with perpendicular branches from this bar towards the anterior implants. Digital impressions were made five times for each type using three intraoral scanners (IOSs). For conventional method, silicone impressions and verification jigs were prepared; fabricated plaster models were scanned using a laboratory scanner/industrial 3D scanner. In analysis 1, two-way ANOVA analyzed the effect of IOSs and assistive devices on the precision of digital impressions. In analysis 2, one-way ANOVA compared the silicone impressions, the verification jigs, and the most precise group of digital impressions from analysis 1.

**Results:**

In analysis 1, the IOS and assistive device type (*F* = 25.22, *p* < .0001) effects and the interaction between these two factors (*F* = 5.64, *p* = .0005) were statistically significant. In analysis 2, CON, VJ, and digital impression with Type 2 devices (most precise devices in analysis 1) were compared; better precision was obtained by digital impression with Type 2 device than by CON and VJ (*F* = 30.08, *p* < .0001).

**Conclusions:**

For implants placed in an edentulous maxilla, digital impressions with assistive devices can provide better precision compared to silicone impressions and verification jigs.

## Background

Digital impressions with intraoral scanners (IOSs) have been rapidly penetrating dental practice in recent years and play a central role in digital dentistry. This enables dental practitioners to transfer patients’ intraoral architecture to digital data without a series of steps such as impressions with elastic materials, plaster model fabrication, and scanning using dental laboratory scanners. It also eliminates the materials and equipment required for the conventional method, such as open trays, silicone impression materials, impression copings, and implant analogs. Therefore, digital impressions benefit implant treatment more than the treatment for natural teeth in terms of time efficiency and resource savings [1].

Screw-fixed implant-supported prostheses cannot compensate for dimensional discrepancies because the buffering effect provided by the periodontal ligament cannot be expected, and the cement space available in cement-fixed prostheses is not present. Therefore, screw-fixed implant-supported prostheses require the highest degree of accuracy to secure long-term stability [2]. Katsoulis et al. proposed clinically acceptable thresholds of the misfit in implant prostheses, and suggested thresholds of within 25 µm for a 3-unit bridge, 50 µm for a 4–9-unit fixed prosthesis, and 50–100 µm for a complete arch implant prosthesis, while a misfit of more than 100 µm for any type of implant prosthesis was deemed unacceptable [3].

Unlike the conventional method, in which dimensional changes of impression materials and plaster can cause distortion, digital impressions are theoretically free of these distortions because IOSs directly capture the intraoral architecture, converting it to digital data using a software [4–6]. In fact, many studies have reported that the accuracy of digital implant impressions for cases with single or few missing teeth is equal to or superior to that of conventional methods [7–9].

In contrast, it has also been reported that the accuracy of digital implant impressions in completely edentulous cases is inferior to that of conventional techniques or is clinically unsatisfactory [9–13].

This can be attributed to the IOSs’ unique process of constructing three-dimensional (3D) images. IOSs build up intraoral images consecutively by stitching together small pieces of the captured images. Consequently, the broader the image captured by the IOS, the larger the accumulated errors from stitching, which was demonstrated by our previous study that reported a decline in the precision of digital impressions as the scanned area was extended [9].

In addition, a greater degree of error is anticipated for impressions of implants placed in a vast edentulous space than for those placed in partially edentulous spaces; this can be attributed to the morphological difficulty caused by flat edentulous mucosa and sparsely-standing and height-varied scan bodies [14]. Kim et al. reported an improvement in the accuracy of digital impressions by placing a projection on the edentulous mucosa [12]. An in vitro study by Mizumoto et al. investigated modified scanning techniques, including mucosal surface modification using glass beads or pressure-indicating paste and scan body splinting technique using dental floss [15].

Hence, connecting the scan bodies with an assistive device with complex morphology and including them into digital impressions may reduce the error caused by the flat edentulous mucosa, thereby improving the precision of digital impressions in cases with extended edentulous space. In this study, assistive devices were developed based on this hypothesis and their effect on the precision of digital impressions for models of the edentulous maxilla with four implants was investigated. The null hypothesis of this study was as follows: Assistive devices do not affect the precision of digital impressions for four implants in an edentulous maxilla, and there is no difference in the precision of digital and conventional impressions.

## Methods

### Reference model fabrication

The reference model was fabricated with type IV dental stone (NEW FUJIROCK IMP, GC, Tokyo, Japan) and implant replicas (Abutment Replica Multi-unit Brånemark System RP, Nobel Biocare, Kloten, Switzerland), which emulated a completely edentulous maxilla with implants (NobelSpeedy Groovy, Nobel Biocare, Kloten, Switzerland) and abutments (Multi-unit Abutment Brånemark System RP, Nobel Biocare, Kloten, Switzerland) at positions #15, #12, #22, and #25. All digital impressions were made by a dentist having more than 3 years of experience with digital impressions in routine practice.

### Fabrication of assistive device

**Fig. 1:**
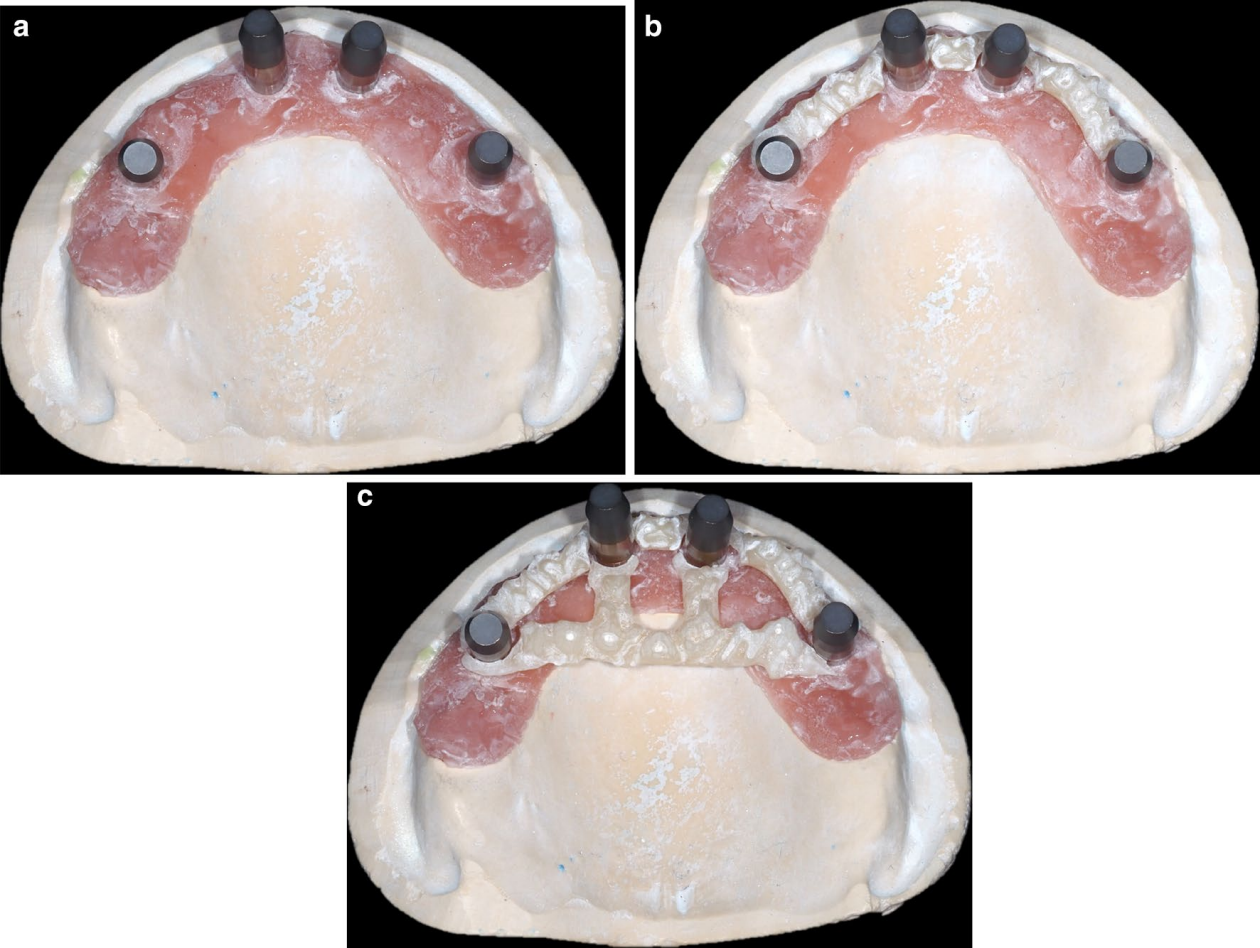
Designs of the assistive devices in the digital impression group. **a** Type 0: Reference model with attached scan bodies, without an assistive device. **b** Type 1: Reference model with an assistive device connecting only neighboring scan bodies, namely between #12–15, #12–22, and #22–25. **c** Type 2: Reference model with assistive device and the same connections as Type 1, along with a connection between two posterior implants and two perpendicular branches towards the two anterior implants

Scan bodies (Position Locator Multiple Nobel Biocare Multi-unit Abutment, Nobel Biocare, Kloten, Switzerland) were connected to the implant analogs in the reference model (Fig. 1a). The model was scanned using a dental laboratory scanner (D810, 3Shape, Copenhagen, Denmark, D810). On this surface data, the assistive device was designed using a CAD software (3Shape, Copenhagen, Denmark).

The Type 1 assistive device connected only the neighboring scan bodies, namely #12-15, #12-22, and #22-25, respectively (Fig.1b).

The Type 2 assistive device connected the neighboring scan bodies, similar to the Type 1 device. However, it also had a connecting bar between the two posterior implants (#15-25), and two perpendicular branches from this bar extended towards the two anterior implants (Fig. 1c). These two types of designs were input to a CAM device (250i, imes-icore GmbH) and milled from a Polymethyl methacrylate disk (M-PM disk, Shofu, Tokyo, Japan).

### Impression making

#### Conventional impression

Conventional silicone impressions were made from the reference model using impression copings and an open tray at 24 °C. Impression copings were connected using a 2.35 mm diameter cobalt–chromium metal wire and fixed by self-curing acrylic resin (FIXPEED, GC, Tokyo, Japan). Twenty-four hours after making the impression, implant analogs were connected to the impression copings on impressions, followed by fabrication of the plaster models. This procedure was repeated and five plaster models were prepared. To acquire the surface data of these models, scan bodies were connected to the implant analogs, and titanium oxide powder (CEREC Opti Spray, Sirona, Long Island City, NY, USA) was sprayed on the surface to inhibit light reflection. They were then scanned using a dental laboratory scanner (D810, 3Shape, Copenhagen, Denmark) and five STL datasets of the conventional impression group were acquired (hereon referred to as “CON”).

#### Digital impression

Three different IOSs were evaluated in this study: Primescan (PS; Dentsply Sirona, USA), 3M True Definition scanner (TDS; 3M ESPE, Seefeld, Germany), and TRIOS scanner 3 (TR; 3Shape, Copenhagen, Denmark). When using TDS and PS, titanium dioxide powder was sprayed on the reference model according to the manufacturers’ recommendations. The digital impression was made according to the manufacturers’ instructions, except for the scan path, which was kept consistent irrespective of the type of IOS used.

This procedure was conducted for models without an assistive device, or “Type 0,” and on models with “Type 1” and “Type 2” assistive devices. The assistive devices were fixed to scan bodies with self-curing acrylic resin (UNIFAST III, GC) to prevent dislodgement by IOSs during the scan.

**Fig. 2:**
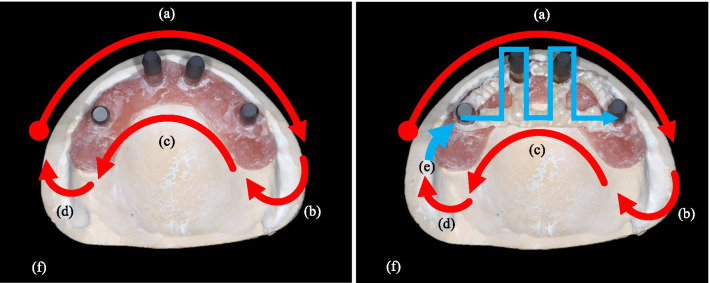
Scanning path of intraoral scanners (IOSs) in each type of reference model. **a** Scanning path in Type 0, 1 **b** Scanning path in Type 2. **a** Scanning with IOS started from the bucco-occlusal surface of the scan body of #15. The bucco-occlusal surfaces were continuously scanned until the scan body of #25. **b** Posterior to the scan body of #25, the scanner was turned from the buccal side to the palatal side. **c** From the scan body of #25, the palatal and occlusal surfaces were scanned continuously until scan body #15. **d** Posterior to the scan body of #15, the scanner was turned from the palatal to the buccal side. **e** The branches of the assisting device on the palate were scanned continuously. **f** A rescan was performed to complement the missing dataset of the first scan

For Type 0 and Type 1, the digital impression started from the bucco-occlusal surface of the #15 scan body, continued along with the bucco-occlusal surface on the alveolar ridge until the #25 scan body, and turned to the palatal and the palato-occlusal surface of the alveolar ridge; scan bodies were scanned continuously until the #15 scan body (Fig. 2a).

For the Type 2 assistive device, branches running over the palate were scanned after scanning along the arch as for Types 0 and 1 (Fig. 2b). Although the operator tried to perform a continuous scan, rescanning was done to complement the missing dataset captured during the first scan. This procedure was repeated five times for each IOS, and all the captured images were converted to the STL format.

### Conventional plaster model via verification jigs

Five verification jigs made of cobalt–chromium alloy were fabricated to fit the reference model. The impression copings were connected to the reference model, and the verification jig was fixed with a self-curing acrylic resin. The verification jig was removed after 30 min, and the laboratory analogs to the impression copings were connected to the impression coping, and the verification plaster models were fabricated, which are commonly considered the most accurate three-dimensional reference in the conventional analogue workflow. The scan bodies were connected to these verification models and scanned using a non-contact 3D measuring instrument (COMET5, Stanbekeley, Germany; hereon referred to as “VJ”) with a reported precision of 1.5 μm.

### Data analysis and evaluation of precision

**Fig. 3:**
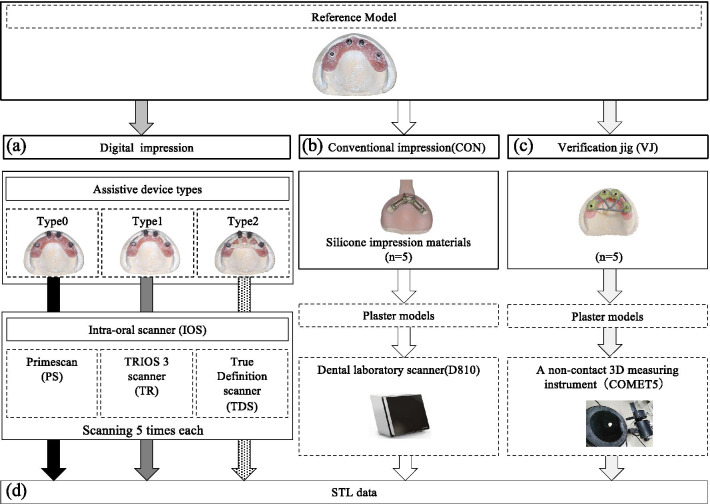
Graphical representation of the overall workflow of this study. **a** Digital impressions were taken five times by all the intraoral scanners in each type. **b** Five conventional impressions with silicone impression materials were obtained, and working casts were fabricated. Five plaster models were scanned by a dental laboratory scanner, and STL datasets were generated. **c** The impression copings were connected to the reference model, and the plaster model was removed after the verification jig was fixed with self-curing acrylic resin. Five plaster models were scanned by a non-contact 3D measuring instrument, and STL datasets were generated. **d** All STL data sets obtained from each group were trimmed and confined to the images of the scan bodies

The overall workflow of this study is presented in Fig. 3. The STL datasets obtained from each group were imported into the measurement software (PolyWorks Inspector, PolyWorks Japan, Tokyo, Japan). Subsequently, only the surface data of the four scan bodies were retained, and the other data were removed.

To evaluate the precision, all combinations of the five STL datasets obtained from each IOS were superimposed using the least-squares best-fit method. Absolute values of the closest distance between the pairs of polygon data were computed for all the surfaces and averaged to determine the surface deviation of the two surface data [6, 9, 16]. To qualitatively evaluate the surface deviation, a color-coded gradient was generated for each situation.

### Statistical analysis

Two types of statistical analyses were performed: analysis 1 compared the type of IOS (PS, TR, and TDS) and types of assistive devices (Type 0, 1, and 2) in relation to their effects on digital impression precision as evaluated by the average deviation (surface deviation) for each situation. Analysis 2 compared the average surface deviation between CON, VJ, and the digital impression group that was the most precise in Analysis 1.

For analysis 1, a two-way analysis of variance was performed, and for analysis 2 a one-way analysis of variance was performed. Tukey's multiple comparison test was used for the post-hoc tests. All statistical analyses software (JMP, SAS Institute Japan, Tokyo, Japan). The level of statistical significance was set at 5%.

## Results

**Fig. 4:**
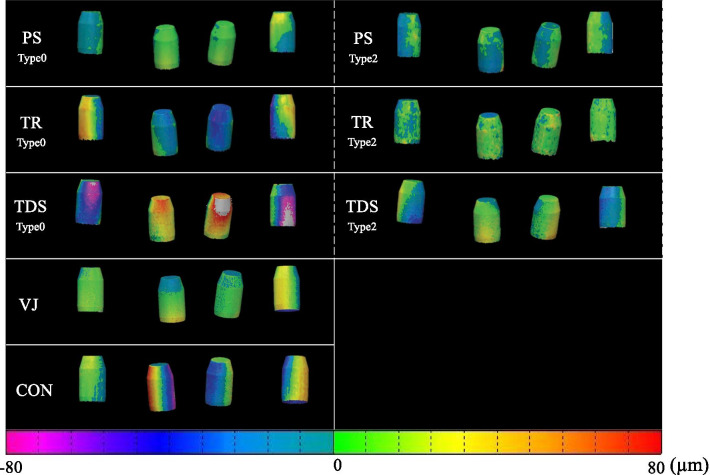
Color-coded deviation maps of surface deviation. They were produced by superimposing two STL datasets through the best-fit algorithm in each IOS for Type 0, 2, and VJ, CON. *CON* conventional impression, *IOS* intra-oral scanners, *VJ* verification jig

**Fig. 5:**
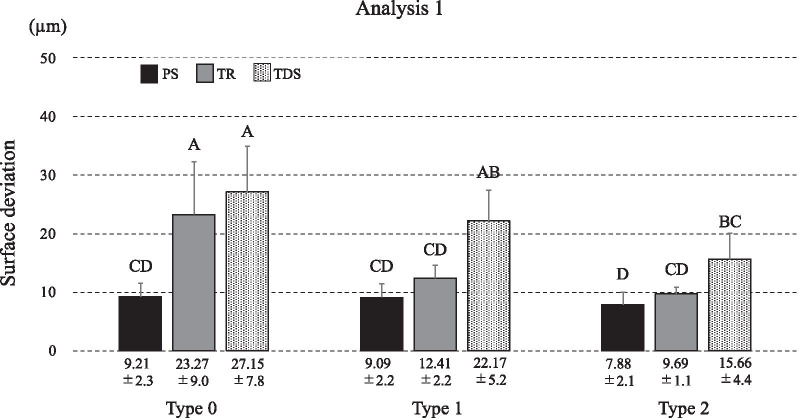
Average discrepancy in each type for each IOS in analysis 1 (µm). The lines on the bars of the bar graph indicate the standard deviation of the measured discrepancies. *IOS* intra-oral scanners, *PS* Primescan, *TDS* true definition scanner, *TR* TRIOS3 scanner

**Fig. 6:**
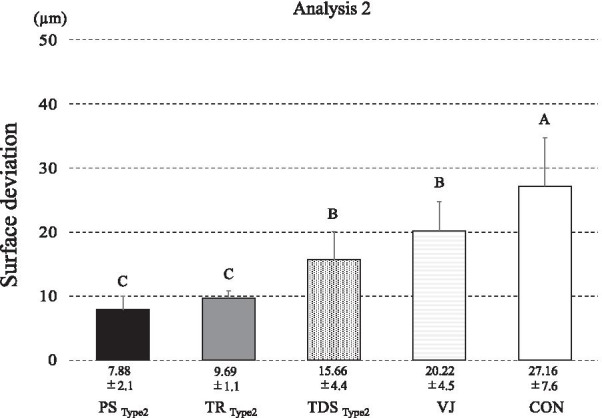
Average discrepancy in each impression method in analysis 2 (µm). The lines on the bars of the bar graph indicate the standard deviation of the measured discrepancies. *PS* Primescan, *TDS* true definition scanner, *TR* TRIOS3 scanner

The numbers of acquired polygon points were as follows: 12068 in CON, 12710 in VJ, 31345 in PS, 18237 in TDS, and 41973 in TR. Figure 4 shows a typical example of the color-coded gradient of surface deviation between superimposed STL data obtained from “PS,” “TR,” “TDS” with each of Type 0, 2, “CON,” and “VJ.” The results of Analysis 1 and 2 are shown in Figs. 5 and 6, respectively.

Analysis 1 revealed that the main effects of “IOS models” (*F*=53.56, *p*<.0001) and “assistive device type” (*F*=25.22, *p*<.0001) were both statistically significant.

The interrelation between the two factors was also statistically significant (*F*=5.64, *p*=.0005) (Fig. 5).

Given that Type 2 showed the best precision in analysis 1, analysis 2 compared the results of the digital impression for Type 2 scanned by three IOSs, CON, and VJ. The effect of impression methods on the precision of impression was statistically significant (*F*=30.08, *p*<.0001; Fig. 6).

## Discussion

### Main findings

In this study, the effect of an assistive device was evaluated in terms of the impact on the precision of digital impression for multiple implants placed in the edentulous maxilla. The use of assistive devices tended to improve the precision of the digital impression to varying degrees depending on the IOSs. In particular, digital impressions with the Type 2 assistive device showed higher precision than those with VJ and CON. Therefore, the null hypothesis of this study was rejected.

Precision of digital impressions of implants in edentulous patient models

### Effect of difference in IOS system

Existing model-based experiments with completely edentulous implant cases have shown that the impression accuracy varies with the type of IOS. Mangano et al. [17] compared the precision of four IOSs (Trios2, CS 3500, Zfx Intrascan, and PlanScan) on a model of the edentulous maxilla with six implants by superimposing three scans each with a best-fit algorithm. The differences were 55.2±10.4 µm in CS3500, 67.0±32.2 µm in Trios, 112.4±22.6 µm in Zfx Intrascan, and 204.2±22.7 µm in PlanScan [17].

In a later study by the same authors, the same method was used to evaluate five different IOSs (Trios3, CS 3600, Emerald, Omnicam, and DWIO), and the results showed that Trios3 had the highest precision (35.6 ± 3.4 µm), followed by CS 3600 (35.7 ± 4.3 µm), Emerald (61.5 ± 18.1 µm), Omnicam® (89.3 ± 14 µm), and DWIO (111 ± 24.8 µm) [18].

Imburgia et al. also compared the precision of four IOSs (CS3600, Trios3, Omnicam, and TDS) by superimposing five scans by each IOS in a model with six implants in the edentulous maxilla. The results showed that Trios3 had the highest precision of 31.5 ± 9.8 µm, followed by Omnicam (57.2 ± 9.1 µm), CS3600 (65.5 ± 16.7 µm) and True Definition (75.3 ± 43.8 µm), without being statistically significant [19].

The authors' previous study evaluated how the impression range affects the precision of digital impressions in an edentulous maxilla model with six implants. The precision of digital impression for the complete arch was 28.6±10.0 µm in Trios2, 21.3±6.1 µm in CCS, 16.4±5.3 µm for TDS, and 18.7±1.4 µm for Omnicam [9]. In this report, better precision was reported than in the other similar studies mentioned above. This is presumably because the authors' previous study restricted the region of interest to the scan body, while the other similar studies set the entire impression as the region of interest for calculating the difference value. In the present study, which exclusively investigated the scan body region as our previous study, precision without the assistive device (27.15±7.8 µm in TDS, and 23.27±9.0 µm in TR) was comparable to the previous study results, except for that of PS (9.21±2.3 µm), which is discussed in the next section.

### Comparison of precision with conventional impression

Earlier studies have compared the precision of conventional impressions with digital impressions. The precision of digital impressions reported in these studies is statistically inferior to the precision obtained by the conventional method and verification jig [10–13]. However, in this study, PS (PS0: 9.21±2.3 µm, PS2: 7.88±2.1 µm) showed better precision than CON: 27.16±7.6 µm and VJ: 20.22±4.5 µm.

It is assumed that the improvements in technology and algorithms and increase in the amount of information processed by IOS might lead to good precision despite the completely edentulous situation, which has been challenging for previous IOSs owing to the aforementioned difficulty in the stitching process. In addition, because the tip of the scanner is larger than the other IOSs, the number of pixels and area that can be captured per frame might be larger.

### Characteristics and effect of the assistive device

Without the assistive devices, the precision of the digital impression was inferior to that of CON and VJ, except for PS. Inadequate precision of digital implant impression for extended edentulous space has also been reported in other studies [8, 9].

Several studies have attempted to solve this problem by placing landmarks on the edentulous ridge to improve the accuracy of digital impressions and to expand the application range of IOSs.

Kim et al. placed an artificial landmark in the center of the edentulous space between #34 and #36 (26 mm interval) on a model of a partially edentulous mandible and showed significant improvement in precision and trueness of digital impressions by Trios, Cerec Omnicam, and CS3500 [12].

Itturate et al. conducted a model-based study simulating four implants placed in the edentulous maxilla, similar to the present study. They placed an assistive device mimicking natural dentition between four impression copings and evaluated the trueness and precision of digital impressions with Trios3, True Definition Scanner, and Itero Element [20]. The results showed that an assistive device improved trueness and precision of digital impressions, but they did not compare with the conventional method.

Huang et al. investigated digital impressions made by Trios3 with an assistive device that connected the scan bodies with each other in comparison with the conventional method using edentulous mandibular model with four implants [21]. The results showed that the precision of the digital impression was improved by the assistive device but was still inferior to that of the conventional method.

These studies attempt to minimize the errors caused by image stitching, which is more likely to occur on the flat edentulous ridge compared to the dentate ridge with a complex topography, using assistive devices placed on the crest as landmarks between the scan bodies [12]. Another major cause of error in edentulous implant cases is the long scanning length in a horseshoe shape between both ends [9]. Therefore, this study designed an additional linear connection between scan bodies at both ends, which added a shorter scanning pass. In fact, significantly better precision was obtained for Type 2 compared to Type 1 and even better than VJ, which is used as a definite dimensional reference for final prosthesis fabrication by conventional methods. While this method required additional scanning, it is speculated that the scanning software algorithm can perform dimensional correction of impression data by incorporating the additional scan data of the linear shortcuts crossing the palate.

Overall, the results of this study suggesting that the precision of digital impression can be improved by an assistive device with a complex topography on edentulous ridge are in line with previous studies. Furthermore, this study demonstrated for the first time that the precision of the impression was improved by adding linear connecting structures between bilateral scan bodies at both ends; this should be regarded as the uniqueness and strength of this study.

### Clinical implications

This study found that the precision of the digital impressing data captured by commercially available IOSs differed according to the model. However, by adding the assistive device described in this study the precision of the digital data for implants placed in edentulous maxilla was greater than that achieved by the conventional method, which was consistently found to be independent of the IOS type used.

### Study limitations

This study only evaluates the precision of the digital impressions, which indicates the closeness between the different test results; trueness, which indicates a closeness to the actual dimensions, was not considered. Thus, a high precision does not guarantee the fit of the prosthesis. Specifically, clinical relevance can only be evaluated by measuring the fit of fabricated prosthesis. We suggest that future studies should investigate the fit of the final prosthesis, which is affected by the entire treatment and manufacturing process, in vivo, if preliminary studies such as ours demonstrate clinically acceptable precision and trueness.

## Conclusion

The results of this study showed that an assistive device significantly improved impression precision, but the effect varied depending on the IOS and type of assistive device. Precision of the digital impression with the Type 2 assistive device was better than that with the conventional method. The Type 2 device connected adjacent implants and had a connecting bar between the two posterior implants (#15–25); in addition, two perpendicular branches to this bar extended towards the two anterior implants. These results suggest that the precision of digital impressions in edentulous implant cases can be improved by the newly developed assistive device, which exceeds the precision achieved by conventional impressions.

## Data Availability

Not applicable.

## Abbreviations

CON: Conventional impression
IOS: Intra-oral scanners
PS: Primescan
TDS: True definition scanner
TR: TRIOS3 scanner
VJ: Verification jig
